# Motor practice related changes in the sensorimotor cortices of youth with cerebral palsy

**DOI:** 10.1093/braincomms/fcae332

**Published:** 2024-09-26

**Authors:** Max J Kurz, Brittany K Taylor, Elizabeth Heinrichs-Graham, Rachel K Spooner, Sarah E Baker, Tony W Wilson

**Affiliations:** Institute for Human Neuroscience, Boys Town National Research Hospital, Boys Town, NE 68010, USA; Center for Pediatric Brain Health, Boys Town National Research Hospital, Boys Town, NE 68010, USA; Department of Pharmacology and Neuroscience, College of Medicine, Creighton University, Omaha, NE 68178, USA; Institute for Human Neuroscience, Boys Town National Research Hospital, Boys Town, NE 68010, USA; Center for Pediatric Brain Health, Boys Town National Research Hospital, Boys Town, NE 68010, USA; Department of Pharmacology and Neuroscience, College of Medicine, Creighton University, Omaha, NE 68178, USA; Institute for Human Neuroscience, Boys Town National Research Hospital, Boys Town, NE 68010, USA; Center for Pediatric Brain Health, Boys Town National Research Hospital, Boys Town, NE 68010, USA; Department of Pharmacology and Neuroscience, College of Medicine, Creighton University, Omaha, NE 68178, USA; Institute for Human Neuroscience, Boys Town National Research Hospital, Boys Town, NE 68010, USA; Institute for Human Neuroscience, Boys Town National Research Hospital, Boys Town, NE 68010, USA; Institute for Human Neuroscience, Boys Town National Research Hospital, Boys Town, NE 68010, USA; Center for Pediatric Brain Health, Boys Town National Research Hospital, Boys Town, NE 68010, USA; Department of Pharmacology and Neuroscience, College of Medicine, Creighton University, Omaha, NE 68178, USA

**Keywords:** neuroimaging, lower extremity, motor learning, physical therapy, frog game

## Abstract

The altered sensorimotor cortical dynamics seen in youth with cerebral palsy appear to be tightly coupled with their motor performance errors and uncharacteristic mobility. Very few investigations have used these cortical dynamics as potential biomarkers to predict the extent of the motor performance changes that might be seen after physical therapy or in the design of new therapeutic interventions that target a youth’s specific neurophysiological deficits. This cohort investigation was directed at evaluating the practice dependent changes in the sensorimotor cortical oscillations exhibited by youth with cerebral palsy as a step towards addressing this gap. We used magnetoencephalography to image the changes in the cortical oscillations before and after youth with cerebral palsy (*N* = 25; age = 15.2 ± 4.5 years; Gross Motor Function Classification Score Levels I–III) and neurotypical controls (*N* = 18; age = 14.6 ± 3.1 years) practiced a knee extension isometric target-matching task. Subsequently, structural equation modelling was used to assess the multivariate relationship between changes in beta (16–22 Hz) and gamma (66–82 Hz) oscillations and the motor performance after practice. The structural equation modelling results suggested youth with cerebral palsy who had a faster reaction time after practice tended to also have a stronger peri-movement beta oscillation in the sensorimotor cortices following practicing. The stronger beta oscillations were inferred to reflect greater certainty in the selected motor plan. The models also indicated that youth with cerebral palsy who overshot the targets less and matched the targets sooner tended to have a stronger execution-related gamma response in the sensorimotor cortices after practice. This stronger gamma response may represent improve activation of the sensorimotor neural generators and/or alterations in the GABAergic interneuron inhibitory–excitatory dynamics. These novel neurophysiological results provide a window on the potential neurological changes governing the practice-related outcomes in the context of the physical therapy.

## Teaser

Kurz *et al.* examined practice-related changes in the sensorimotor cortical oscillations serving isometric knee extensions in youth with cerebral palsy and demographically matched controls. They showed that motor performance after practice was associated with a stronger reduction in sensorimotor beta oscillations and an increase in the sensorimotor gamma oscillations during movement.

## Graphical Abstract

**Graphical Abstract:**
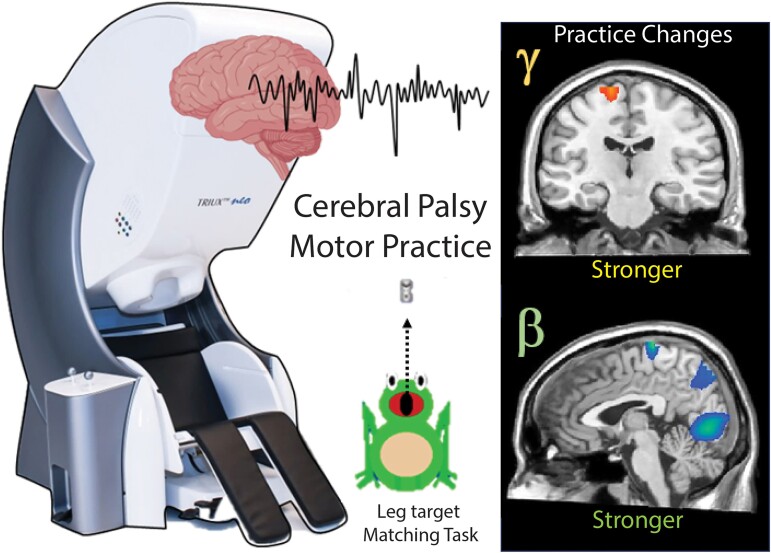

## Introduction

Cerebral palsy (CP) is an umbrella term that is used to describe the clinical presentation following a brain injury that occurs *in utero* or shortly after birth.^1^ The brain injuries can arise from a myriad of factors (i.e. stroke, placenta abruptions, infections, respiratory distress, etc.) that often provoke a cascade of sensorimotor impairments that impact motor function.^2^ There have been numerous physical therapies that have been employed to help children with CP better control their leg motor actions and improve mobility.^3,4^ However, the evidence for these approaches are mixed with some children with CP demonstrating beneficial improvements while others appear to be non-responders. This response variability represents a primary Gordian knot for the advancement of physical therapy protocols used in youth with CP.

Most of the clinical literature focusing on lower extremity impairments in youth with CP has centred on improving the muscle strength or altering the muscular architecture via surgical treatment approaches.^5-8^ This premise assumes that the motor control problems primarily reside in the musculoskeletal machinery^9^ and does not fully consider the role the sensorimotor cortices in the regulation of the muscle force production. Recognizing this shortcoming, modern physical therapy trends have shifted their focused towards alterations in the neurophysiology of motor control in youth with CP and how this may impact the fidelity of the motor actions.^10,11^ Several magnetoencephalographic (MEG) brain imaging studies have revealed that there are clear aberrations in the beta and gamma cortical oscillations while youth with CP plan and execute motor actions.^12-17^ Specifically, youth with CP exhibit stronger beta oscillations (i.e. decreases in power relative to baseline or beta desynchronization) during the motor planning and execution stages compared to controls. Such changes in the strength of the beta desynchronization are thought to reflect the extent of the disinhibition of the resting sensorimotor cortical synchrony in order to facilitate motor performance.^18-24^ As such, the stronger beta sensorimotor cortical oscillations observed in youth with CP is currently thought to reflect the recruitment of a larger population of neurons for the planning and execution of motor actions after practice. Moreover, sensorimotor gamma oscillations have been shown to be weaker in youth with CP when executing the motor command. This weaker gamma event-related synchronization (ERS) has been interpreted to be due to the limited number of tracts that can be excited due to the corticospinal tract damage.^25^ Lastly, the extent of alterations in the sensorimotor cortical dynamics have been shown to be linked with slower reaction times, overshooting of target forces, and extended time to match targets when youth with CP perform a lower extremity isometric target-matching task.^12-17^

Despite these neurophysiological insights, very few investigations have leveraged these cortical oscillations as potential biomarkers to predict the extent of motor performance changes that might be seen after physical therapy or to inform the design of new therapeutic interventions so that they target specific neurophysiological deficits. A recent study aimed to begin to fill this knowledge gap by examining whether the strength of motor-related beta oscillations were altered after persons with CP completed a physical therapy protocol that involved performing weighted squats on a sled machine as fast-as-possible over an 8 week period.^12^ The outcomes from this seminal investigation showed that the strength of beta event-related desynchronizations was weaker in the sensorimotor cortices and more like the neurotypical (NT) controls after undergoing the physical therapy protocol. Furthermore, these changes in the strength of beta oscillations were linked to improvements in the ability to generate muscular power. Although these initial insights were insightful, we still have a limited understanding of the multivariate relationship between practice dependent changes in gamma and beta sensorimotor cortical oscillations and motor performance changes seen in youth with CP.

Several prior investigations have quantified the practice dependent changes seen in NT controls. PET and fMRI investigations have shown that the primary motor area, supplementary motor area, prefrontal cortex and parietal cortex exhibit changes in the strength of activation after participants practice a motor task.^26-32^ These cortical changes were associated with improved spatial processing, sensorimotor transformations, online error corrections and improved resource allocation.^33-36^ Prior MEG studies have also shown that the strength of sensorimotor beta oscillations during motor planning and execution are weaker following practice of an isometric force matching task, with complementary changes in motor performance that include faster reaction times and improved accuracy.^37,38^ These authors proposed that the alterations in beta cortical oscillations potentially reflect a more refined motor plan or a reduction in neural resources needed to perform the motor task. Surprisingly, none of the outcomes from these key studies have been extended to advance our understanding of how practice impacts the altered sensorimotor cortical neurophysiology seen in youth with CP.

Overall, there are still notable gaps in our understanding of the neurophysiological changes induced by the physical therapy practice paradigms being used in youth with CP. Continuing to pursue this knowledge gap has the potential to clarify which treatment protocols have the best potential to improve the planning and execution of leg motor actions in youth with CP. Thus, in the current investigation, we directly evaluated practice dependent changes in the beta and gamma cortical oscillations that underlie movement in youth with CP. To this end, we used high-density MEG to quantify changes in motor-related oscillations before and after youth with CP and NT controls practiced a knee extension isometric target-matching task. Subsequently, structural equation modelling (SEM) was used to assess the multivariate relationship between practice dependent changes in beta and gamma cortical oscillations and the extent of changes in motor performance. We hypothesized that before practicing the youth with CP would have slower reaction times, would be less precise in matching targets, and would take more time to match the targets when compared with the NT controls. However, after practice, the youth with CP would demonstrate improvements in the respective motor behavioural variables. We also hypothesized that a reduction in the strength of sensorimotor beta oscillations during the motor planning and execution stages would be linked with the youth with CP having a faster reaction time, less overshoot of the targets, and faster matching of the targets. Lastly, we also hypothesized that stronger sensorimotor gamma oscillations after practice would also be tightly connected with the motor performance of the youth with CP after practice.

## Materials and methods

### Subjects

This cohort investigation was conducted at a research hospital and the Institutional Review Board approved the investigation. Consent was obtained according to the Declaration of Helsinki where the guardians provided written informed consent, and the youth assented to participate. Youth with CP included in this investigation not had an orthopaedic surgery or anti-spasticity treatments within the last 6 months, or metal in their body that would preclude the use of MEG. Furthermore, none of the participants with CP had significant brain volume loss that would affect the integrity of the cortical surface. The NT controls included in this investigation had no known neurological or musculoskeletal impairments. Using the smallest effect size (Cohen’s *d* = 1.34) seen in the motor practice paradigm performed by NT controls in Gehringer et al.,^37^ nine participants would provide >80% power to detect a similar behavioural performance improvement at a 0.01 alpha level.^37^ To make the outcomes more generalizable, this investigation collected evaluable pre–post-practice data from 43 participants. Twenty-five of the participants had CP with a spastic diplegic presentation (age = 15.2 ± 4.5 years; gross motor function classification score (GMFCS) levels I–III; 15 male), and 17 of the participants were NT controls (age = 14.6 ± 3.1 years; 10 male).

### Magnetoencephalographic data acquisition and experimental paradigm

**Figure 1:**
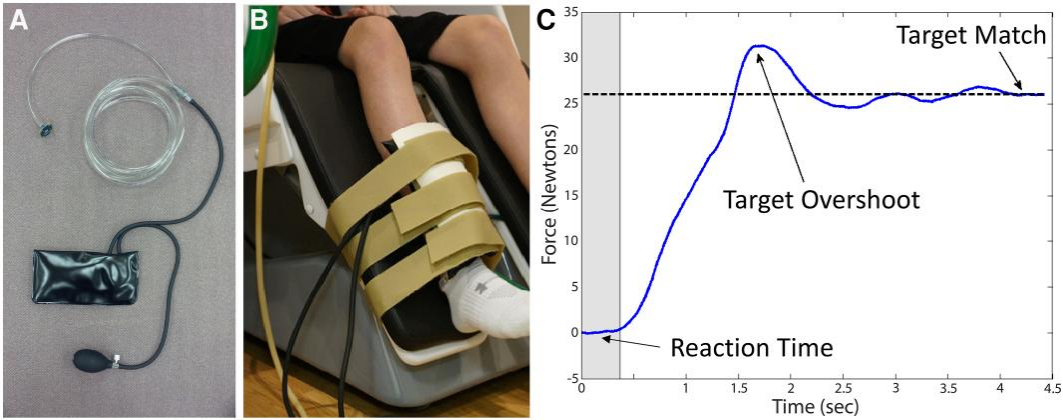
**Pneumatic force transducer.** (**A**) Components of the pneumatic force transducer that consisted of an inflatable airbag that was connected to a pressure sensor via tubing. The tubing was fed through a waveguide in the magnetically shielded room and into a component rack that contained the pressure sensor. The pressure sensor was subsequently connected to an analogue-to-digital board. (**B**) Depiction of the positioning of the pneumatic force transducer for measuring the knee extension isometric forces while the participant underwent magnetoencephalography. (**C**) Exemplary output of the pneumatic force transducer when matching a target. The targeted is graphically depicted on the post-analysis data as a dashed line. The behavioural outcome variables of reaction time (greyed area), percent target overshoot and time to match the target are shown.

All recordings were conducted using a whole head MEG system (MEGIN/Elekta, Helsinki, Finland) that was in a one-layer magnetically shielded room with active shielding engaged for advanced environmental noise compensation. The neuromagnetic responses were sampled continuously at 1 kHz with an acquisition bandwidth of 0.1–330 Hz. A custom-built, magnetically silent pneumatic force transducer was used to measure the isometric knee extension forces generated by the participants (Fig. 1).^12,14,16^ This device consisted of air bladder (20 × 10 cm) that was inflated to 317 kPa and was integrated within a thermoplastic shell that was secured to the seat leg pedals via Velcro strapping. Changes in the pressure of the airbag as knee extension forces were generated, measured by an air pressure sensor (Phidgets Inc., Calgary, Alberta, CA, USA) and were converted into force units offline. This system has been reliably used across numerous MEG neuroimaging investigations.^12,14,37-39^

**Figure 2:**
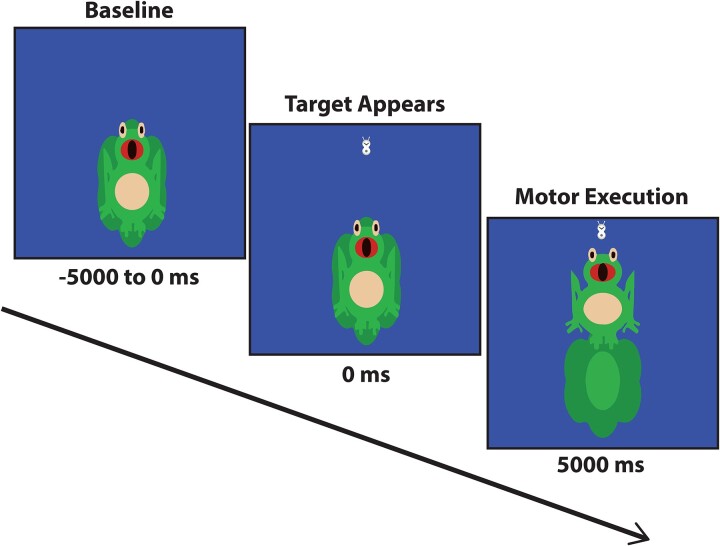
**Experimental paradigm.** For each trial, the generated isometric force animated the frog cartoon vertically as the participant attempted to position the target bug in the frog’s mouth. The bug’s position varied randomly and was between 15 and 30% of the participant’s baseline isometric force. The participants initially performed 100 target-matching trials while undergoing MEG (baseline). Subsequently, they practiced 100 trails while receiving visual and verbal feedback on how to improve their motor performance. Lastly, the participants underwent MEG again and performed 100 additional targets.

The experimental paradigm involved the participant generating isometric knee extension forces with their right leg to match target forces that were between 15 and 30% of the participant’s maximum isometric force. The target force was visually displayed as a bug, and the force generated by the participant was shown as a frog that animated vertically based on the amount of isometric force (Fig. 2). The participants were instructed to match the presented targets as fast and as accurately as possible. The target forces were presented in a random order, and a successful match occurred when the bug that represented the target force was inside the frog’s mouth for 300 ms. The stimuli were shown on a back-projection screen that was ∼1 m in front of the participant and at eye-level. Each trial was 10 s in length. The participants started each trial at rest for 5 s. The target bug appeared after the rest period and the participant generated an isometric force that matched the force value as quickly and as accurately as possible. The target bug was available to be matched for up to 5 s. Once the target was matched or 5 s elapsed, feedback was given to indicate the end of the trial. The participant subsequently returned to the rest position while waiting for the next target bug to appear. Participants performed three blocks of the target-matching task, with each block containing 100 trials. All practice blocks were performed during a single session, with a 5 min break between each block. Hence, the total time for the experiment was ∼60 min. The first and third blocks were performed during the MEG recording, while the second block was an intensive practice block. During the intensive practice block, the participant was provided information about the accuracy of their target-matching performance via an interactive biofeedback program that showed how far the participant overshot the target. Hence, all participants received the same knowledge of the performance from the biofeedback program. We augmented the interactive program by providing verbal feedback on how to improve the motor performance. This feedback was individualized and often centred on not overshooting the target and focusing on generating a precise isometric force that would match the target. Subsequently, when the participant reduced the amount of overshoot, we focused on encouraging the participants to match the targets as quickly as possible while still not overshooting the target. As expected, the controls were able to modify their motor performance more rapidly based on the interactive feedback and verbal coaching. In this case, they quickly relied on the interactive program for self-monitoring their motor performance.

During the MEG recordings, a video camera was used to monitor the participant’s engagement in the task and their behavioural performance was shown on a computer monitor in the control room. Intermittent verbal encouragement via the intercom system was provided if it was qualitatively perceived that the participant’s engagement was reduced. However, this was rarely the case for the participants included in this investigation.

### Magnetoencephalographic pre-processing and source imaging

Prior to the experiment, four coils were affixed to the participant’s head and the location of the coils, three fiducial points, and the scalp surface were digitized (Fastrak 3SF0002, Polhemus Navigator Sciences, Colchester, VT, USA). During the MEG recording, an electric current with a unique frequency label (e.g. 322 Hz) was fed to each of the coils and was used to localize the head in reference to the MEG sensors. The participant’s MEG data were subsequently co-registered with the MRI and transformed into standardized space.

Each participant’s MEG dataset were individually corrected for head motion that occurred during task performance using the MaxFilter software (MEGIN/Elekta). In addition, the signal space separation method with a temporal extension was used for noise reduction.^40^ All of the MEG data pre-processing, co-registration and source imaging was performed with Brain Electrical Source Analysis (BESA) software (BESA v6.0; Grafelfing, Germany). Artefact rejection was based on an individualized fixed threshold method and supplemented with visual inspection. The continuous magnetic time series were divided into epochs of 10.0 s in duration (− 5.0 s to +5.0 s), with the onset of the isometric force defined as 0.0 s and the baseline defined as − 2.0 to − 1.4 s. Artefact-free epochs for each sensor were transformed into the time-frequency domain using complex demodulation and averaged over the respective trials. These sensor-level data were normalized to the mean power during the baseline, and the specific time-frequency windows selected for source imaging were determined by statistical analysis of the sensor-level spectrograms across the entire array of gradiometers from all participants and practice blocks.^41-43^ Based on these time-frequency windows, a minimum variance vector beamformer was used to calculate the source power across the entire brain volume per participant at a 4.0 mm^3^ resolution.^44^ The source power in these images were normalized per subject using a separately averaged pre-stimulus noise period of equal duration and bandwidth.^45,46^

The peak voxels identified in the grand-averaged beamformer images of each oscillatory response across both groups were used for extracting virtual-sensor neural time series data per participant during the pre- and post-practice data collections. The virtual sensors were computed by applying the sensor weighting matrix derived through the forward computation to the pre-processed signal vector.^43,47^ Once the virtual sensors were extracted, they were transformed into the time-frequency domain, and the two orientations for each peak voxel per individual were combined using a vector-summing algorithm. The minima (in the case of beta responses) or maxima (in the case of gamma responses) change in the neural time series across the window of interest was then used to assess practice dependent changes in the key oscillatory responses seen in the whole brain beamformer images.

### Motor behavioural data

The output of the pneumatic force transducer was simultaneously collected at 1 kHz along with the MEG data and was used to quantify the motor performance. The formulation of the motor plan was assumed to be represented by the participant’s reaction time, which was calculated based on the time from when the target was presented to when force production was initiated. The reaction time was assumed to represent the participant’s conceptualization and preparation to perform the motor action.^48,49^ The amount of error in the feedforward execution of the motor plan was quantified based on the per cent overshoot of the target. The time to match the target was used to quantify the online corrections that were made after the initial motor plan was executed. The online corrections were calculated based on the time difference between the reaction time and the time to reach the target. The average performance on each of these metrics across the respective trials was used to quantify each participant’s motor performance.

### Statistical analysis

Initially, separate 2 × 2 mixed-model ANOVAs (pre-/post-practice × group) were performed for the respective motor performance measures to clarify that the participants did demonstrate behavioural practice dependent effects. We subsequently used a multi-group SEM to disentangle the multivariate relationship between practice dependent changes in cortical oscillations and motor performance. The SEM modelling we employed does resemble a traditional multiple regression approach. However, we specifically utilized a multi-group SEM approach, which allowed us to simultaneously model these predictive multivariate relationships uniquely for our NT and CP groups, thereby reducing the number of tests performed while still informing us about important differences in the strength and directionality of associations between identified neural markers and critical aspects of behavioural performance on the task. Specifically, the model used in this investigation included changes in the beta and gamma cortical dynamics (e.g. beta local minima during pre-practice MEG session—beta local minima during post-practice MEG session) as continuous predictors of the respective motor performance measures seen after practice. Additionally, correlations between the disparate cortical dynamics were modelled as shared covariance in the model structure. Fisher-*z post hoc* was used to evaluate if the strength of the identified significant coefficients were different for the CP and NT models. Separate SEMs were created for the reaction time, target overshoot and time to match the target. All models were examined for goodness of fit using standard criteria,^50^ including root mean square error of approximation < 0.06, comparative fit index > 0.95, and standardized root mean square residual < 0.08. All models and statistical analysis were conducted using R and the Lavaan software package. In the text, *β* represents the standardized coefficient and *b* represents the unstandardized coefficient.

## Results

### Motor behavioural results

Overall, our results showed that participants improved in their ability to accurately match the prescribed targets. For reaction time, there was a practice block main effect (Pre: 553 ± 26 ms, Post: 567 ± 34 ms; *F* = 126.4; *P* < 0.001), indicating that all participants had moderately slower reaction times after practice. There was also a significant group main effect (CP: 606 ± 32 ms, NT: 490 ± 24 s; *F* = 6.33; *P* = 0.016) showing that in general the youth with CP had slower reaction times when compared with the controls. The interaction term was not significant (*F* = 0.75; *P* = 0.391).

For the target overshoot, there was not a practice block main effect (Pre: 14.7 ± 2.5%, Post: 12.9 ± 2.8%; *F* = 2.48; *P* = 0.123), suggesting that there was not an appreciable change in the amount of overshoot from pre- to post-practice. However, there was a group main effect (CP: 19.7 ± 3.0%, NT: 5.1 ± 0.72%; *F* = 10.95; *P* = 0.016), signifying that the youth with CP tended to overshoot the targets more compared to the controls. The interaction term was not significant (*F* = 0.80; *P* = 0.804).

For the time to match the targets, there was a practice block main effect (Pre: 2950 ± 121 ms, Post: 2483 ± 116 ms; *F* = 21.61; *P* < 0.001), showing that all participants tended to match the targets faster after practice. There was also a group main effect (CP: 3015 ± 126 ms, NT: 2278 ± 85 ms; *F* = 15.30; *P* < 0.001), signifying that the youth with CP tended to take longer to match the targets overall. The interaction term was not significant (*F* = 0.38; *P* = 0.542).

### Cortical oscillations at the sensor and source level

**Figure 3:**
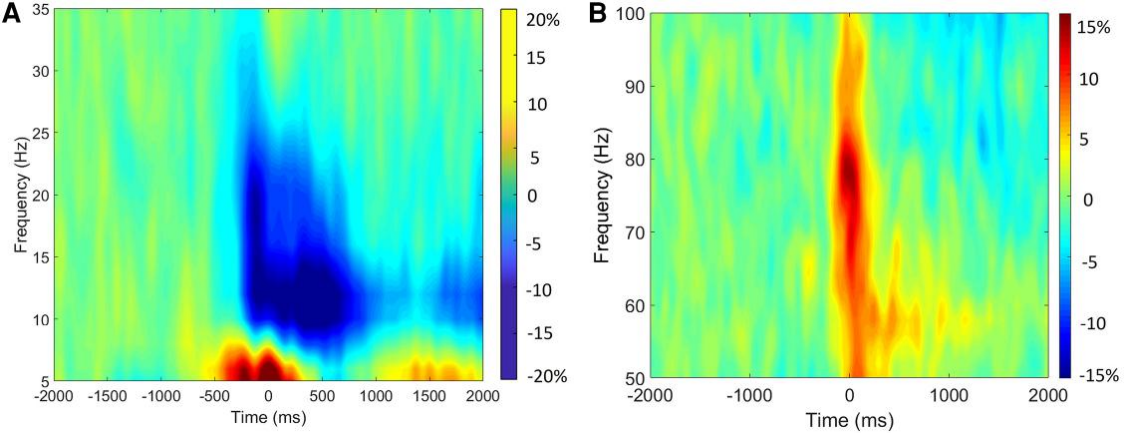
**Motor-related oscillations.** Time-frequency spectrograms grand averaged across both groups and practice time points. Frequency (Hz) is shown on the *y*-axis and time (ms) is denoted on the *x*-axis, with 0 ms defined as the onset of the isometric force. The time-frequency spectrogram of the MEG sensor with the greatest response amplitude is shown, which was located near the sensorimotor cortices contralateral to the (right) leg generating the force. The colour bar represents per cent change in power relative the baseline (**A**) A beta band (16–22 Hz) ERD was detected in MEG sensors near the sensorimotor cortex. This response started about 300 ms prior to the initiation of the isometric force and persisted during the muscular contraction (∼900 ms). (**B**) A gamma (66–82 Hz) ERS was also noted in the same sensors and was tightly yoked with the onset of the movement.

Visual inspection of the grand-averaged oscillatory responses showed that there were notable changes in the gradiometers that spanned the Rolandic (i.e. sensorimotor cortices) and occipital regions. Statistical analysis of the sensor-level time-frequency spectrograms revealed significant oscillatory activity in the beta (16–22 Hz) and gamma (66–82 Hz) frequency ranges (*Ps* < 0.0001, corrected; Fig. 3). Specifically, the sensor-level statistical analyses indicated that there was a prominent beta oscillatory response [i.e. event-related desynchronization (ERD), or decrease in power from baseline] that began 300 ms prior to the onset of the isometric force (i.e. 0 ms) and was sustained for ∼900 ms (i.e. −300–900 ms; Fig. 3A). Furthermore, there was a prominent gamma response (i.e. ERS, or increase in power from baseline) that was transient and tightly yoked to the onset of the isometric force (i.e. 0–100 ms; Fig. 3B).

**Figure 4:**
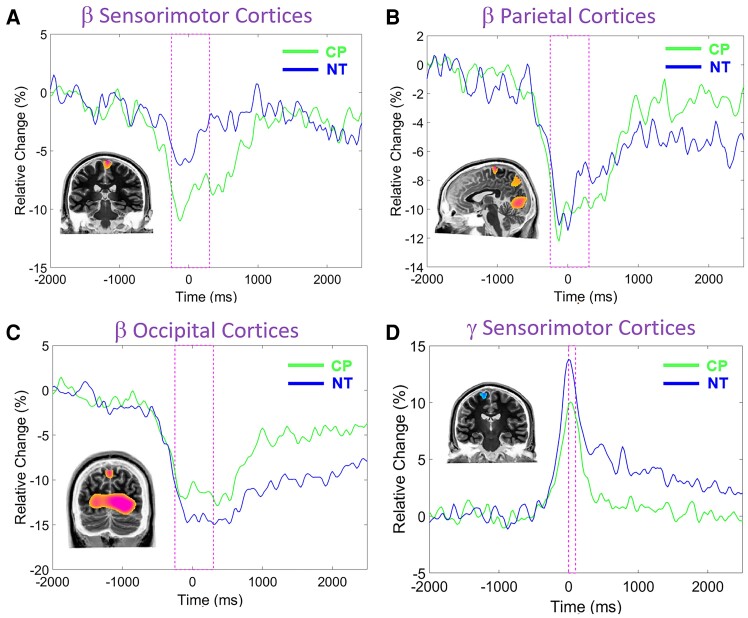
**Source images and neural time series.** Each panel displays the grand-averaged source image and group averaged neural time series extracted from the peak voxel of the respective images across both pre- and post-practice. The dashed area on the neural time series identifies the time window that was imaged, while the lighter lines represent the neural response seen in the participants with CP and the darker line represents the response seen in the NT controls. As shown, the time series of the sensorimotor (**A**), parietal (**B**) and occipital (**C**) beta ERD responses across the motor planning and execution stages. Further, the gamma ERS within the sensorimotor cortices (**D**) that was closely yoked with the muscular force onset. Qualitatively, the neural time series for the sensorimotor beta ERD was stronger for the youth with CP when compared with the NT controls. The neural time series for the beta ERD in the occipital cortices was also weaker for the youth with CP. Lastly, we observed that the gamma ERS was weaker for the youth with CP when compared with the NT controls. CP, cerebral palsy and NT, neurotypical control.

The beta ERD across the −300 to 300 ms time window, and gamma ERS across the 0 to 100 ms time window were subsequently imaged using a beamformer. Of note, we focused on the −300 to 300 ms time window for the beta response to maximize the signal-to-noise ratio, as the response was much stronger during that window. The resulting grand average maps indicted that the beta ERD was generated by neural populations in the leg region of the contralateral sensorimotor cortices (Fig. 4A), with additional clusters seen in the parietal (Fig. 4B) and occipital cortices (Fig. 4C). The grand average maps also showed that the gamma ERS was in the leg region of the sensorimotor cortices (Fig. 4D). The neural time courses were extracted from the peak voxels per participant, cluster and practice block for the respective cortical oscillations and subsequently used for the SEM models. Given that we did not have laterality hypotheses or use a task that would generate hemispheric-specific activity, the peak voxel in the right hemisphere of the occipital cortices was used since it was the strongest in the grand-averaged image. For display purposes, the average neural time series for the youth with CP and NT controls are also shown in Fig. 4. These time series are consistent with previously reported literature, in that the youth with CP exhibited stronger beta ERD responses in the leg region of the sensorimotor cortices (Fig. 4A) and a weaker beta ERD in the occipital cortices (Fig. 4C). Furthermore, the neural time courses suggest that there was a weaker gamma ERS for the youth with CP when compared with the NT controls (Fig. 4D). The respective local minima and maxima of the neural time series from the practice block conditions were subtracted (i.e. pre-practice block − post-practice block) and used in the SEMs that evaluated the multivariate relationship between the practice dependent changes in cortical oscillations and performance.

### Structural equation models

**Figure 5:**
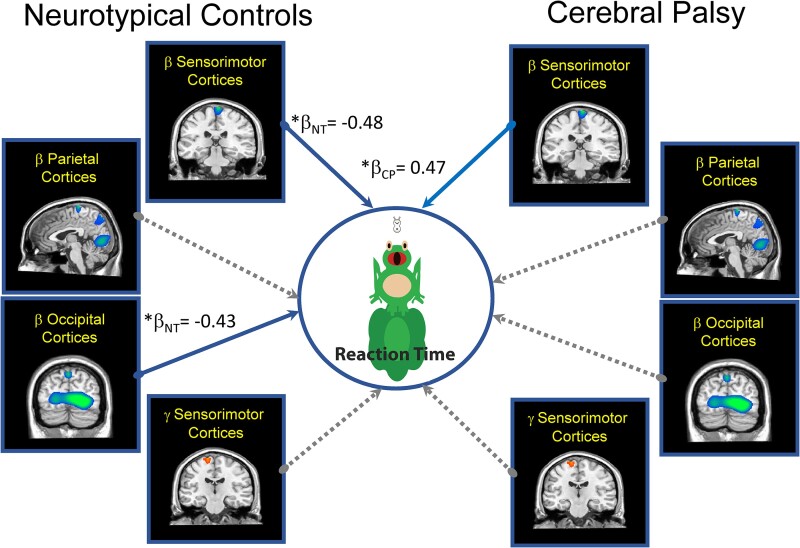
**Practice dependent changes in the cortical oscillations that predict reaction time.** Summary of the main effects seen in our structural equation model of the multivariate relationship between practice dependent changes in the strength of the beta ERD, gamma ERS and the post-practice reaction time. Solid lines represent significant predictive paths, while the dashed lines indicate non-significant paths. The significant standardized coefficients are shown. Overall, the model suggested that weaker beta ERD responses in the sensorimotor (*β*_NT_ = −0.48, *P* = 0.02) and occipital cortices after practice were associated with faster reaction times for the NT controls (*β*_NT_ = −0.43, *P* = 0.04). This was not the case for the youth with CP (*β*_cp_ = 0.47, *P* = 0.05). For the youth with CP, a stronger sensorimotor beta ERD after practice was associated with a faster reaction time. NT, neurotypical control, CP, cerebral palsy, *P ≤ 0.05.

The SEM that included the reaction time after practice met standards for an excellent fit (Fig. 5; root mean square error of approximation = 0.0001; comparative fit index = 1.00; standardized root mean square residual = 0.0001). In NT controls, the practice dependent change in the beta ERD of the sensorimotor (*b*_NT_ = −1.03; *β*_NT_ = −0.48, *P* = 0.02) and occipital (*b*_NT_= −0.92; *β*_NT_ = −0.43, *P* = 0.04) cortices were significant predictors of reaction time. This implies that the NT controls with a greater post-practice reduction in the strength of the beta ERD (i.e. less negative) also tended to have faster reaction times. For the youth with CP, the practice dependent change in the sensorimotor beta ERD (*b*_CP_ = 1.95; *β*_cp_ = 0.47, *P* = 0.05) was also a significant predictor of reaction time. This suggests that the youth with CP who had a greater post-practice increase in the strength of the beta ERD (i.e. more negative) tended to have a faster reaction time, while those with a weaker beta ERD tended to have a slower reaction time. Our follow-up *post hoc* analyses showed that the respective coefficients were significantly stronger for the NT controls (*Z* = 2.715; *P* = 0.003). Hence, inferring that the degree of the change in the sensorimotor and occipital beta ERD was a better predictor of the reaction times seen in the NT controls after practice.

**Figure 6:**
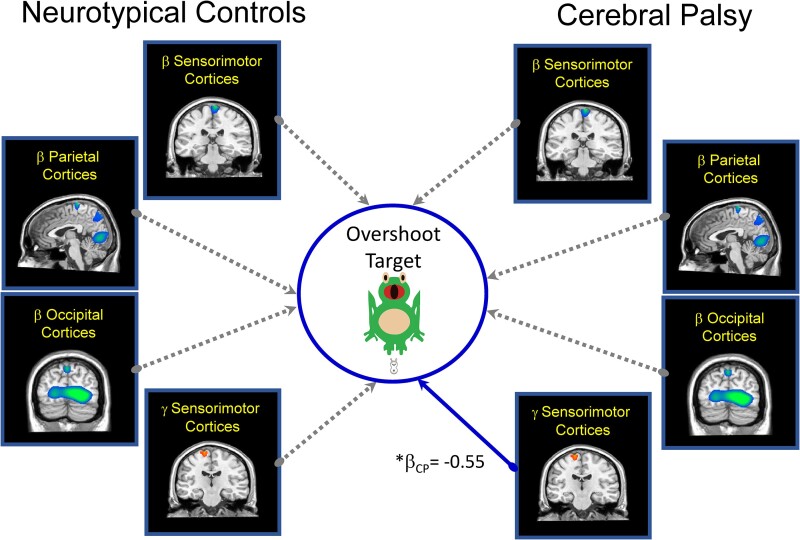
**Practice dependent changes in the cortical oscillations that predict target overshoot.** Summary of the main effects seen in our structural equation model of the multivariate relationship between the practice dependent changes in the strength of the beta ERD, gamma ERS and the amount of overshoot of the targets after practice. Solid lines represent significant predictive paths, while the dashed lines indicate non-significant paths. Overall, the model suggested that a stronger sensorimotor gamma ERS after practice was associated with less overshoot of the targets for the youth with CP (*β*_CP_ = −0.55, *P* = 0.003). CP, cerebral palsy, *P ≤ 0.05.

The SEM that included the amount of overshoot of the targets after practice met standards for an excellent fit (Fig. 6; root mean square error of approximation = 0.0001; comparative fit index = 1.00; standardized root mean square residual = 0.0001). For the youth with CP, the practice dependent change in the sensorimotor cortical gamma ERS (*b*_CP_ = −10.14; *β*_CP_ = −0.55, *P* = 0.003) was a significant predictor of the amount the targets were overshot after practice. This implies that the youth with CP who had a stronger gamma ERS after practice also tended to be more accurate, with less overshoot of the targets. In contrast, there were no practice-related changes in the neural markers that predicted behaviour in NT controls. Our follow-up *post hoc* analyses showed that the sensorimotor gamma ERS coefficient was significantly stronger for the youth with CP when compared with the NT controls (*Z = −*2.357; *P* = 0.0092). Hence, implying that the degree of the change in the sensorimotor gamma ERS for the youth with CP was a better predictor of the amount the targets were overshoot after practice.

**Figure 7:**
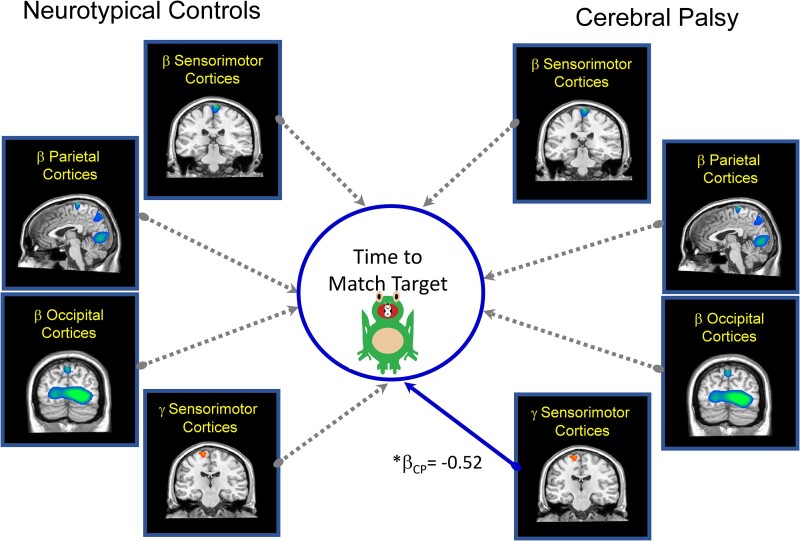
**Practice dependent changes in the oscillations that predict time to match target.** Summary of the main effects seen in our structural equation model of the multivariate relationship between the practice dependent changes in the strength of the beta ERD, gamma ERS and the time to match the target after practice is shown. Solid lines represent significant predictive paths, while dashed lines indicate non-significant paths. The significant standardized coefficients are shown. Overall, the model suggested that a stronger sensorimotor gamma ERS after practice leads to matching the targets sooner for the youth with CP (*β*_CP_ = −0.52, *P* = 0.003). CP, cerebral palsy, *P ≤ 0.05.

The SEM that included the time to match the targets after practice met standards for an excellent fit (Fig. 7; root mean square error of approximation = 0.0001; comparative fit index = 1.00; standardized root mean square residual = 0.0001). For the youth with CP, the practice dependent change in the sensorimotor cortical gamma ERS (*b*_CP_ = −3.90; *β*_CP_ = −0.52, *P* = 0.003) was a significant predictor of the time it took to match the targets after practice. This implies that the youth with CP who had a stronger gamma ERS after practice also tended to match the targets sooner. In contrast, there were no practice-related changes in neural markers that predicted behaviour in NT controls. Our follow-up *post hoc* analyses suggested that the sensorimotor gamma ERS coefficient was not definitively stronger for the youth with CP when compared with the NT controls (*Z* = −0.738; *P* = 0.230).

## Discussion

There is mounting evidence that shows youth with CP have aberrant sensorimotor cortical oscillations that are connected with their uncharacteristic motor performance. Despite this evidence, there have been limited efforts to explore how these cortical oscillations change after practicing a motor task. In this investigation, we explored the relationship between changes in beta and gamma cortical oscillations and motor performance after youth with CP practice an isometric knee extension target-matching task. Our hypotheses were confirmed in that youth with CP had slower reaction times, were less precise in generating isometric forces to match targets and took more time to match the targets before practicing the target-matching task when compared with the NT controls. However, the interaction terms (Group X Pre-/Post-Practice) for the respective motor behavioural variables were not significant suggesting that there were individual differences in the practice-related effects. We subsequently employed SEM to assess the multivariate relationship between the practice dependent changes in beta and gamma cortical oscillations and the extent of individual improvements in the motor performance. Our hypothesis that the SEM would show that a reduction in the strength of the sensorimotor beta ERD after youth with CP practice would be associated with a faster reaction time was refuted. However, our hypothesis that a stronger gamma sensorimotor ERS after practice would be tightly connected with the motor performance of the youth with CP was accepted. Further discussion of these key outcomes and clinical translation are presented in the following sections.

There is now substantial evidence that the sensorimotor beta ERD is uncharacteristically stronger in youth with CP and is connected with the extent of the motor performance errors.^12-17^ Our results add to this body of literature by showing that reaction times seen in youth with CP after practicing a knee isometric task are moderated by the extent of practice dependent changes in the sensorimotor beta ERD. The behavioural results indicated that youth with CP that had a faster reaction time tended to have a stronger sensorimotor beta ERD after practice. The beta desynchronization is thought to reflect the extent of the disinhibition of the resting sensorimotor cortical synchrony in order to facilitate motor performance.^18-24^ As such, the stronger beta ERD seen in this study might reflect the recruitment of more neurons for the planning and execution of the motor action after practice. A stronger beta ERD has also been linked with greater certainty in the selected motor action.^51^ Therefore, the current data might imply that the youth with CP have greater certainty in selecting an isometric muscular force that would match the presented targets.

Conversely, a weaker beta ERD after practice was associated with a faster reaction time for the NT controls. This directly contrasts to what was seen for the youth with CP, where we suggested that the stronger beta ERD was associated with greater certainty in the selected isometric force production. We speculate that this simply reflects that the task was not as challenging for the controls, as they likely have mastered the isolation and selection of the single joint movement and thus, the changes in beta activity following practice may be more related to fine-tuning the movement rather than improvement, *per se*. Nevertheless, we infer that the weaker beta ERD suggests that NT controls recruit fewer neurons for the planning and execution of the motor action after practice. This premise concurs with the numerous neuroimaging studies (e.g. fMRI, EEG and PET) with NT controls that have shown that the amount of sensorimotor cortical activity is reduced or optimized after practicing a novel motor task.^32,52-60^ In addition, the reduced cortical activity after practice may also indicate that NT controls need less cognitive resources to successfully perform the motor task.^34,35,37,38^ Alternatively, the nature of the practice dependent changes seen in the sensorimotor cortical oscillations might be fundamentally different for youth with CP, as they have incurred a brain injury. While this is unlikely future studies are needed to fully exclude this possibility.

Another key finding of this investigation was that a stronger gamma ERS after practice was associated with the youth with CP overshooting the targets to a lesser degree and matching the targets faster. Prior research has identified that the gamma ERS is weaker for youth with CP when compared with controls^13,16^ and have speculated that the reduced strength of the oscillations is related to the corticospinal tract damage seen in youth with CP.^25^ The results shown here suggest that practice might result in the recruitment of more sensorimotor cortical neurons for improvement in the corticospinal output when generating isometric muscular forces that meet the task demand. Alternatively, they might suggest that practice affects the excitatory and inhibitory balance of the sensorimotor cortical neurons. Prior MEG studies have provided supporting evidence that the concentration of the inhibitory GABA neurotransmitter within the cortices appears to be linked with the strength and frequency of local gamma oscillations.^61,62^ Furthermore, PET studies have also shown that children with CP tend to have increased GABA receptor binding potential within the sensorimotor cortices.^63,64^ Based on this evidence, we speculate that the stronger gamma ERS seen in the youth with CP after practice might also be partly a result of altered GABA activity.

Finally, it should be noted that the generation of an isometric knee extension force involves an integration of the activity seen in the cortical and spinal cord neural generators.^65^ Essentially, the spinal cord alpha motoneurons receive direct input from the corticospinal tracts and represent the final stage of muscular force production. Thus, another interpretation is that the stronger motor-related gamma ERS seen after practice reflects improved coherence between the output of the sensorimotor cortex and the spinal cord alpha motor neurons. These practice dependent neurophysiological changes would be beneficial, as there is mounting evidence that CP is associated with structural changes in the grey and white matter composition of the spinal cord^17,66^ and that there are alterations in the fidelity of the spinal cord interneuronal dynamics while producing an isometric muscular force.^67,68^ As such, the increased sensorimotor gamma oscillatory activity might reflect an increased ability to excite the alpha motor neurons for the generation of a precise muscular contraction after practice. Although this speculation is plausible, this premise needs to be further evaluated to fully disentangle the potential concurrent changes in the sensorimotor gamma ERS and the spinal cord dynamics with practice.

Although this experimental work has provided unique insights on the practice dependent neurophysiological changes seen in youth with CP, there are several limitations that should be recognized. For one, this investigation evaluated the influence of a practice session that was performed during a single day, and the long-term implications for learning a motor task remain unknown. To better address the cortical changes associated with motor learning, we would need to reassess the cortical dynamics and motor performance at a later time point. Secondarily, we used a simple isometric force motor task to model the practice dependent changes that might represent what is seen after youth with CP undergo physical therapy. In part, this choice was based on the types of motor actions that can be practiced while undergoing MEG brain imaging. Furthermore, we chose to initially study a simple motor action as the treatment goals and fidelity of physical therapy protocols can be divergent across patients and treatment sessions. Hence, our approach was to reduce the degrees of freedom that generally encompass a physical therapy treatment paradigm in hopes of deriving foundational knowledge. Third, it should be noted that the practice paradigm employed in this study was done in one session and not over several sessions. It is possible that the changes seen in the strength of the sensorimotor beta and gamma cortical oscillations might be dose dependent. Lastly, this investigation was very motor centric and was unable to fully address how the somatosensory processing deficits seen in youth with CP also influenced the practice dependent changes.^69,70-73^ An inability to interpret the ongoing sensory feedback likely impacts the ability to alter the motor plan and/or internal model of the expected outcomes of the feedforward motor action. Despite these limitations, the practice dependent changes in the cortical dynamics highlighted by our experimental work provides unique insight on the multifaceted roles of the beta and gamma oscillations on the motor planning and execution process in youth with CP.

## Supplementary Material

fcae332_Supplementary_Data

## Data Availability

The data are available upon reasonable request to the corresponding author. The R code used for the respective models is provided in the Supplementary material.
