# Supplementary material for: Motor practice related changes in the sensorimotor cortices of youth with cerebral palsy
Source: Brain Commun. 2024 Sep 26;6(5):fcae332. doi: 10.1093/braincomms/fcae332 (PMC11465084; doi:10.1093/braincomms/fcae332)
Supplement: fcae332_Supplementary_Data [file fcae332_supplementary_data.pdf]

subject	Group	Post_RT	Post_Targetmatch	Post_Overshoot	PrePost_Change_Beta_Motor
39	0	0.398667	2.607466667	7.794146667	-0.0962
41	0	0.945533	3.811133333	30.43909333	-0.02854
43	0	0.370067	1.388733333	2.40734	0.05167
44	0	0.637	3.029866667	6.41974	-0.0475
45	0	0.673533	3.3212	6.130566667	-0.0134
47	0	1.007533	3.9924	69.69715333	0.0526
48	0	0.948333	1.814866667	1.894166667	-0.016
49	0	0.374333	4.092333333	22.08879333	-0.0375
50	0	0.539933	2.9896	13.2024	-0.0328
52	0	0.443467	2.431066667	3.112626667	-0.0029
53	0	0.567867	2.4252	6.9695	-0.03433
54	0	0.367	2.2862	5.53104	0.0459
55	0	0.5498	2.4096	20.65616667	0.0309
57	0	0.427667	3.421866667	31.58949333	-0.0544
59	0	0.438	2.262133333	4.605133333	-0.0091
62	0	0.545333	2.077266667	5.5934	0.0339
64	0	0.376133	2.677533333	14.29391333	-0.1147
71	0	0.480867	3.4316	16.26916667	0.0488
76	0	0.361	4.251466667	69.11584	-0.0837
80	0	0.493133	2.4976	5.482866667	0.0111
81	0	1.119467	3.6804	17.76945333	0.02393
82	0	0.7898	2.368733333	10.39902667	0.059
84	0	0.478867	3.820133333	45.73818	0.0532
87	0	0.582133	2.724933333	17.24563333	-0.0945
93	0	0.512333	3.321866667	18.83456667	0.0277
65	1	0.487733	2.249133333	0.717586667	-0.0271
67	1	0.429533	1.788933333	2.528306667	0.0369
68	1	0.5874	3.0552	11.48195333	0.0024
69	1	0.489867	2.022733333	3.759326667	-0.0568
70	1	0.406667	1.828533333	2.715073333	-0.01557
73	1	0.479333	1.512266667	2.716186667	-0.0879
74	1	0.377933	1.5058	4.972393333	0.0777
75	1	0.316333	1.845666667	2.368633333	0.0583
77	1	0.4158	2.129066667	1.905653333	0.0067
88	1	0.5754	2.304333333	12.01394	-0.06327
89	1	0.317533	2.771866667	12.26126667	0.0822
90	1	0.372	2.685466667	3.632566667	0.0023
91	1	0.572733	3.0658	6.98768	0.027
94	1	0.3068	2.191266667	5.147573333	0.0017
95	1	0.419533	1.611933333	0.351646667	-0.0742
96	1	0.230933	1.646666667	1.562406667	0.02173
103	1	0.427667	2.444466667	4.187126667	-0.04827
104	1	0.6304	0.759466667	7.027993333	0.0343

PrePost_Change_Beta_Parietal	PrePost_Change_Beta_Visual	PrePost_Change_Gamma
-0.0506	-0.0516	0.0045
-0.0077	-0.053915	-0.00948
0.09197	0.082615	0.0446
-0.0316	-0.052485	0.02943
0.00987	-0.03925	-0.021
-0.0015	-0.0057	-0.2202
0.0142	0.0203	0.22779
-0.0139	-0.0695	0.01564
-0.0172	-0.12285	-0.06701
0.0332	-0.0277	-0.07159
0.0624	0.007165	-0.0243
0.1415	0.0627	0.1201
-0.0013	0.02155	0.014
-0.0414	0.0021	0.0625
0.0418	-0.05315	0.032153
-0.052	-0.02815	0.14973
-0.15807	0.047615	-0.00525
-0.0009	0.11855	0.05479
0.0039	-0.00505	-0.02805
0.0049	0.0009	-0.05254
-0.0018	0.011785	-0.01781
0.0568	-0.011	0.0306
0.019	0.042515	-0.129
0.069	-0.0456	0.079
0.0224	-0.03955	-0.2444
0.05253	0.01665	0.0057
0.1242	0.0434	0.02552
0.0814	-0.111665	0.00719
0.09007	0.085	0.01621
0.0452	0.02185	0.0817
-0.02143	-0.00055	0.05294
0.03973	-0.0001	-0.04636
0.0142	0.050485	-0.03819
-0.00453	0.0272	0.0076
-0.0119	0.06115	-0.03179
0.0614	-0.00435	-0.1252
-0.0509	-0.04355	0.0674
0.018	-0.0259	-0.00759
0.0273	-0.01795	0.0176
-0.0101	-0.02125	-0.06976
0.035	0.0707	0.1902
0.06607	0.088435	0.0338
0.0157	-0.03085	0.0646

```
###load library

library(lavaan)

library(semPlot)


#input data

megdata <- read.csv("BrainComm_Dataset.csv")


#setup data

megdata$Group = factor(megdata$Group)

megdata$Post_Overshoot=megdata$Post_Overshoot/10


#Model 1

#model = '

#Post_Overshoot~  PrePost_Change_Beta_Motor + PrePost_Change_Beta_Parietal +
PrePost_Change_Beta_Visual + PrePost_Change_Gamma

#PrePost_Change_Beta_Motor~~PrePost_Change_Beta_Parietal

#PrePost_Change_Beta_Motor~~PrePost_Change_Beta_Visual

#PrePost_Change_Beta_Motor~~PrePost_Change_Gamma

#PrePost_Change_Beta_Parietal~~PrePost_Change_Beta_Visual

#PrePost_Change_Beta_Parietal~~PrePost_Change_Gamma

#PrePost_Change_Beta_Visual~~PrePost_Change_Gamma

#'



#Model 2

#model = '

#Post_RT~  PrePost_Change_Beta_Motor + PrePost_Change_Beta_Parietal +
PrePost_Change_Beta_Visual + PrePost_Change_Gamma

#PrePost_Change_Beta_Motor~~PrePost_Change_Beta_Parietal
```

```
#PrePost_Change_Beta_Motor~~PrePost_Change_Beta_Visual

#PrePost_Change_Beta_Motor~~PrePost_Change_Gamma

#PrePost_Change_Beta_Parietal~~PrePost_Change_Beta_Visual

#PrePost_Change_Beta_Parietal~~PrePost_Change_Gamma

#PrePost_Change_Beta_Visual~~PrePost_Change_Gamma

#'


#Model 3

#model = '

#Post_Targetmatch ~  PrePost_Change_Beta_Motor + PrePost_Change_Beta_Parietal +
PrePost_Change_Beta_Visual + PrePost_Change_Gamma

#PrePost_Change_Beta_Motor~~PrePost_Change_Beta_Parietal

#PrePost_Change_Beta_Motor~~PrePost_Change_Beta_Visual

#PrePost_Change_Beta_Motor~~PrePost_Change_Gamma

#PrePost_Change_Beta_Parietal~~PrePost_Change_Beta_Visual

#PrePost_Change_Beta_Parietal~~PrePost_Change_Gamma

#PrePost_Change_Beta_Visual~~PrePost_Change_Gamma

#'

path = sem (model, data = megdata, group = "Group", missing ="ML")

varTable (path)

summary(path, standardized = T, fit.measures = T, rsquare = T)

semPaths(path, whatLabels="std", layout="tree")
```
